# Experimental, Analytical, and Numerical Assessments for the Controversial Elastic Stiffness Enhancement of CFRP-Strengthened Timber Beams

**DOI:** 10.3390/polym14194222

**Published:** 2022-10-08

**Authors:** Khaled Saad, András Lengyel

**Affiliations:** Department of Structural Mechanics, Budapest University of Technology and Economics, 1111 Budapest, Hungary

**Keywords:** timber, CFRP fabric, in situ strengthening, Norway spruce, tension modulus, compression modulus

## Abstract

The strengthening of timber beams with carbon-fibre-reinforced polymers (CFRP) has been widely used in the last decades to enhance the behaviour of historical or new timber structures, usually for bending. While considerable improvement in capacity and ductility is typically achieved, the increase in stiffness was, in many cases, well short of analytical expectations, which tend to overestimate stiffness. This study addresses the problem by investigating the underlying mechanical behaviour using experimental, analytical, and numerical tools on a sample of Norway spruce (*Picea abies*) beams reinforced with carbon-fibre fabric. In the experimental program, each beam is tested for bending with and without CFRP reinforcement in order to determine specimen-specific stiffness increase on an individual basis. The reinforcement yielded an increase of 27% in capacity, 53% in ultimate displacement, and 133% in compliance, verifying its efficiency. Axial compression tests on an independent sample are also performed to verify modulus of elasticity in compression. Numerical computations based on a beam model and a three-dimensional finite element model are performed with the introduction of separate moduli of elasticity for tension and compression in timber. Inverse computation using the experimental load–deflection curves yielded the moduli and the compression yield stress of timber to provide the best match between tests and simulations. The mean difference of only 6% in stiffness between FEM and the tests is obtained. The dominance of normal stresses in the longitudinal direction is found, in correspondence with the experimentally observed tensile failure of timber (apart from a few defected specimens). Compression yield stresses are within 7% (beam model) and 2% (FEM) error compared with the control axial tests. The differences between FE simulations and tests in ultimate load and compliance are within 1%. This study concludes that the application of CFRP in the composite beams enables the determination of timber material properties opposed to pure timber beams without reinforcement, and the adoption of separate moduli of elasticity for tension and compression leads to adequate modelling of reinforced timber beams.

## 1. Introduction

Wood is a popular building material for a wide range of lightweight structures. Further to its good mechanical properties and simplicity of manufacturing, its aesthetic appearance also makes it a favourable construction material.

The increase of loads or material deterioration in timber structures may sometimes be responsible for the destruction of existing buildings. However, it is often less costly and more time-effective to reinforce damaged wood pieces or structural members rather than replace them entirely. Carbon-fibre-reinforced polymers (CFRP) are currently strongly suggested for structural retrofitting. They have many benefits, including high elastic modulus, strength, corrosion resistance, durability, adequate bonding to wood, and low density. Compared with other types of fibres—e.g., glass or basalt—carbon fibre is more expensive to produce and tends to show brittle failure, though these drawbacks are well counterweighed by numerous advantages. It possesses high stiffness and strength, making it an appropriate alternative to steel in structural reinforcement or completely replacing it, e.g., high-strength steel bridge cables [1]. Its good corrosion resistance makes it a suitable application in extreme environmental circumstances, such as high temperature or pressure [2]. It performs better than fibreglass in low-velocity impact tests as well [3].

Fibre-reinforced polymers are formed by embedding high-strength fibres (carbon, glass, etc.) in a matrix (epoxy, etc.) and can take a range of shapes and sizes (lamella, strip, rod, pultruded elements, etc.), as well as can be prepared on-site or prefabricated. Attachment to wood can usually be externally bonded, near-surface mounted, glued-in, etc. Due to its high modulus and strength, increases in stiffness, capacity, and ductility of reinforced timber structural elements is expected.

The prediction of mechanical behaviour is heavily affected by the uncertainty and variability of material properties of both timber and reinforcement. On the one hand, in the case of CFRP, the manufacturer specifies the properties of fibres. While factory-made composites are available with expected quality and specifications, in situ preparation of the reinforcement always involves uncertainty to a degree. On the other hand, timber quality widely varies due to its natural origin, which is further increased by factors such as moisture content, age, density, species, etc. Measured mechanical properties may show significant differences between samples even from the same species and are frequently only informative [4,5].

The adhesive’s performance directly influences the bond strength and effectiveness of the composite. It is crucial for achieving a robust CFRP-to-wood bond interface that can successfully transmit shear and normal stresses from the timber to the composite material [6]. The bond strength can be affected by a variety of factors (e.g., the properties of wood [7] and the moisture content [8]). Nonetheless, regardless of the characteristics of the matrix, the failure of the timber element was identified as the primary failure mechanism in another investigation, showing that the qualities of the wood, rather than the adhesive, may affect the bonding behaviour [9].

Several experimental studies were carried out to evaluate the effect of CFRP on the flexural behaviour of wood structural components focusing on stiffness, capacity, and ductility. With a good interface between the wood and the CFRP, a significant improvement (maximum of a 60% increase in capacity and a 30% increase in stiffness) was achieved [10]. Two different reinforcement lengths were studied by [11] in three-point bending tests finding that shorter reinforcements enhance stiffness by 23% and ultimate capacity by 12%, compared with longer ones that increase stiffness by 34% and load-bearing capacity by 28%. Sawn Norway spruce beams reinforced with varied quantities of CFRP were experimentally investigated by [12], and they found a 30% improvement in load-bearing capacity and a nearly 16% increase in elastic stiffness. In a similar work, theoretical and experimental analysis on two distinct wood species retrofitted with different amounts of CFRP sheets was performed [13], reporting an increase in load-bearing capability of at least 39%.

It was also shown that, in most cases, strengthening timber elements with CFRP had only a minor influence on stiffness [14,15,16], and the increase is generally not more than 30%. Wood beams from an ancient bridge were strengthened using bidirectional carbon fabrics and laminate strips, resulting in a substantial improvement in shear and bending capacity (40–53%) but only a modest increase in stiffness (17–27%) [17]. A research study employing GFRP dowel bars to strengthen wood stringers found a 70% improvement in ultimate capacity and ductility with a minor increase in elastic stiffness [18]. However, the amount of fibres might be proportionate to the improvement in stiffness [14]. FRP lamella was used for in situ reinforcing to restore and strengthen existing wood parts, reporting a 6% increase in bending stiffness and a 25% increase in flexural strength [19]. A comparable study achieved a 58% increase in load capacity and a minor improvement in beam elastic stiffness [20]. Another work also revealed a 21.5% rise in strength and a slight improvement in stiffness of 4.69% [21]. A 10% increase in stiffness and a 44–60% increase in capacity were observed by [22]. Moreover, the use of CFRP sheets for wood strengthening resulted in a stiffness increase of 15–60% [23], and an increase of 20% in another study [24]. The introduction of carbon plates and sheets leads to a stiffness increase varying from 20.2% to 29.6% [25].

It is apparent that results are scattered in a wide range and sometimes contradict expectations. A review comparison between the measured stiffness increase and the theoretical values obtained from calculations using classical beam theory was shown in [26] based on several research works processed. In some studies, the increase agreed with the theoretical estimates, e.g., [17,27,28], while the results contradicted the theoretical expectations in many other studies, e.g., [23,29,30].

Several numerical and analytical investigations addressed the question. They attributed the differences to an individual factor such as the boundary conditions, the adhesive stiffness, the FRP-to-wood ratio, or the wood material characteristics to resolve the disparities from a technical perspective. For instance, ref. [31] observed a considerable error of 25% between the experimental and the numerical stiffness increase. Ref. [11] restricted the issue to the boundary conditions and parametrically adjusted the penetration coefficient constant, introduced in the FEA software (ANSYS), between the timber and the support plates to anticipate the same experimental stiffness increase. This approach does not resolve the problem of having to set different values in each individual case. Ref. [32] stated that as the fibre percentages increase, the stiffness of the material will most certainly increase. The adhesive materials were also considered the only factor responsible for the differences between the experimental and analytical/numerical applications [33]. The authors suggested considering the cohesive stiffness of the adhesive in the numerical method. As for the wood, it was stated that the CFRP could improve the load-carrying capabilities of a timber beam with a low elastic bending modulus [29].

All the methods mentioned above are strategies to resolve the problem from a technical point of view without explaining the cause of the problem, whereas this work investigates the underlying mechanical behaviour of the materials of CFRP-reinforced timber beams and gives an explanation as to the observed differences between measurements and analysis. For this purpose, a number of sawn Norway spruce beams were tested. Opposed to conventional testing programs, in this work, the bending stiffness properties and enhancement were not obtained as statistics on samples but each specimen was measured with and without unidirectional carbon-fibre fabric reinforcement attached to the tension side in order to obtain a series of specimen-specific data on mechanical properties. Analysis by both classical beam theory and three-dimensional finite element modelling was performed, also capable of predicting the non-linear behaviour by appropriate settings of material parameters. Recommendations concluded by this study on timber modelling enable more adequate assessment of reinforced timber beams in terms of stiffness, preventing possible overestimation of actual stiffness and failing to comply with design objectives.

The outline of the paper is as follows. Section 2 presents the bending tests performed on the sample of beams together with the axial compression test used later for verification of the numerical analysis. Section 3 elaborates on the methods used in this study, including the formulation for the classical beam theory for inhomogeneous beams and the finite element modelling applied as an independent control for the analysis. Section 4 presents the results divided into appropriate parts, including the stiffness values, timber parallel-to-grain moduli of elasticity, observations on capacity and failure, and numerical simulations. The last section summarises the conclusions of this work.

## 2. Experiments

### 2.1. Bending Tests

A series of experiments were carried out for the determination of elastic stiffness of the timber beams with and without reinforcement in order to provide data for the subsequent analysis.

The timber material used is sawn wood of Norway spruce (*Picea abies*). The test beams had a length of approx. 1000 mm (to ensure a clear span of 900 mm) and rectangular cross-sections of approx. 45 mm×45 mm. The moisture content was 12%. The specimens were visually inspected prior to tests to confirm that there were no apparent flaws or damage in the material, such as drying splits, biological degradation, etc. However, knots are considered natural features of the timber and could not be completely eliminated from the sample. The amount of knots and the wood density are known factors that affect the overall modulus of elasticity (MOE), as reported by [34].

In one set of specimens, unidirectional carbon-fibre fabric SikaWrap^®^-231C was combined in situ with epoxy to form reinforcement directly on the surface of the timber beams. The fabric is essentially unidirectional with 99% of fibres in warp. The fabric had a nominal thickness of 0.129 mm, a specific mass of 235 ± 10 g/m${}^{2}$, and a modulus of elasticity 230 GPa, provided by the manufacturer Sika (Baar, Switzerland). The nominal fibre strength and ultimate strain were given as 4900 N/mm${}^{2}$ and 1.7%, respectively.

The adhesive applied is a Sikadur^®^-330 two-component epoxy impregnation resin, also provided by the manufacturer Sika, with a mixing ratio of components equal to 4:1. The density of the mix is 1.30 ± 0.1 kg/m${}^{3}$. The nominal ultimate stress and strain were given as 30 N/mm${}^{2}$ and 0.90%, respectively. According to the product specification on recommended applied amount per unit area, the thickness of the adhesive layer can be between 0.5385 mm and 1.1538 mm. A thicker bond at the contact may generate higher porosity. The appropriate amount of adhesive was portioned for each beam to provide an average thickness of 0.8 mm on the beam surface, which was polished using sandpaper prior to reinforcing.

**Figure 1:**
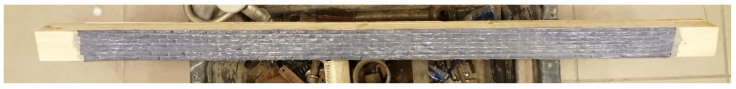
Reinforcing specimens with CFRP fabric.

The contact was made by applying a roller on the surface gently to avoid any damage or unnecessary waviness to the fibres and to ensure a perfect CFRP-to-wood bond interface. An accurately measured amount of epoxy was administered. Note, however, that achieving a uniform adhesive thickness is challenging due to the pressure applied on the surfaces. The fabric was fitted on one face of the beam (tension side) at full width in the clear span leaving space for supports (Figure 1).

**Figure 2:**
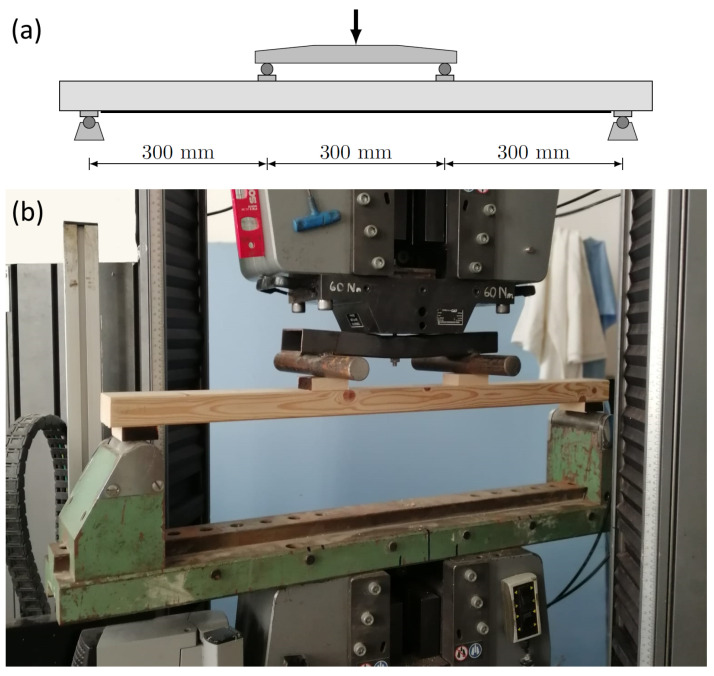
Bending test setup. (**a**) Sketch of loading arrangement; (**b**) testing machine.

A series of four-point bending tests were carried out involving several groups of specimens using a standard MTS testing device with a capacity of 600 kN. The testing arrangement had a clear span of 900 mm, and the force exerted by a single actuator was transmitted onto the specimens at two points at exact thirds of the span via a loading bridge. Small hardwood (beech) blocks were placed at points of application of forces to avoid undesired stress concentration and excessive transverse strains in the softwood specimens. The schematic drawing and a photograph of the test arrangement are shown in Figure 2.

The beams were tested under displacement-controlled loading producing load–deflection data. The loading rate was set to 7 mm/min. The series contained 19 specimens, including 6 without reinforcement (group 0) and 13 reinforced with CFRP fabric (group F), see Table 1.

Before measurements, the physical dimensions and weights of the specimens were measured and documented. The density was computed. (The data are listed together with results in Section 4.) The specimens were devoid of any defects visible on the surface. As knots located in the tension zone could significantly affect the overall mechanical behaviour, especially load-bearing capacity, by the appropriate orientation of the beam, such cases could be avoided in order to prevent premature failure.

During the bending tests, all beams were loaded until the onset of failure. As the process is displacement-controlled, the measurement can continue even if partial damage occurs or negative slopes are obtained. The load–deflection curves are considered terminated at the highest load level, i.e., after which the same load cannot be achieved again. In the case of the reinforced beams, characteristics related to the pure timber are also of interest; therefore, loading tests in the linearly elastic range were performed prior to the application of CFRP. The applied maximum load was carefully set to remain strictly in the elastic range based on the measurement results of a pilot specimen.

### 2.2. Compression Tests

In addition to bending tests, parallel-to-grain compressive tests were also performed in this study to determine the elastic and inelastic properties of timber for modelling. Ten specimens of cross-section 30 mm × 30 mm and length 180 mm were tested. Due to the small size of the specimens, they were selected by screening out pieces containing knots and visible defects. The tests were carried out using an MTS mechanics testing machine according to standard EN 408:2010+A1:2012(E). The procedure was displacement-controlled in order to capture the entire curves.

## 3. Methods

### 3.1. Overview

Data obtained from the experiments form the basis of analytical and numerical investigations. The overall purpose is to evaluate the effect of the CFRP on the bending stiffness of timber beams to make an adequate assessment of the material properties and to provide an accurate prediction for the stiffness increase using analytical and numerical approaches. The individual steps are as follows.

- Stiffness of all tested beams are determined by linear curve fitting to the linear sections of the load–deflection diagrams.
- Considering the classical Euler beam model, equilibrium equations of a homogeneous cross-section (for a non-reinforced beam) and of an inhomogeneous (composite) cross-section (reinforced beam) are formulated in terms of geometric data and the modulus of elasticity for timber and fibre reinforcement. In each measurement pair (same timber beam with and without reinforcement), modulus for timber can be computed from the stiffness of the non-reinforced one, and then the modulus for reinforcement from the other once the modulus for timber is known.
- As in several cases, the obtained modulus for reinforcement is beyond any acceptable tolerance for the factory data provided by the manufacturer, an improved model involving different moduli for tension and compression is introduced, and the equations are reformulated. With the assumption of the factory data for reinforcement, the timber moduli are computed from the equations.
- In order to cross-check the validity of computations based on the Euler beam, a three-dimensional finite element model was constructed involving a fully orthotropic material model and non-linear analysis. The two models are compared for each beam.

The methods are detailed in the following.

### 3.2. Analytical Considerations

All load–deflection curves recorded in the bending tests can be evaluated to obtain bending stiffness characterising linearly elastic behaviour. In the case of loading in the linearly elastic range, the entire curves are used for linear fitting, whereas in the case of total loading (until failure) only the section between 10% and 40% of the ultimate load is considered, where linearity holds. Slope *S* of the line fitted with linear regression relates the increment of applied force *F* and of displacement *e* of the actuator as S=ΔF/Δe.

Considering the classical Euler beam model, some straightforward analytical derivation yields the stiffness of a homogeneous beam under the given test arrangement as $S=64.8EI/L^{3}$, where *E* is the modulus of elasticity (customarily denoted by the acronym MOE), *I* is the moment of inertia related to the axis of bending, and *L* is the span. This allows the computation of the modulus of elasticity from the measurements. It is important to note that the modulus of elasticity is unquestionably the most determining of the material properties of timber beams in terms of displacements. The properties of natural materials vary in a large range, even within the same species. This implies that the calculation of the actual values of MOE for non-reinforced timber beams enables a more accurate evaluation of the same beams with reinforcement than the adoption of statistical averages from literature data or other samples.

Values of modulus of elasticity determined for specimens in group F in the non-reinforced stage are incorporated in the subsequent analysis of reinforcements. The increase in the bending stiffness of reinforced beams is attributed to the contribution of CFRP to the composite structure. Stresses in the inhomogeneous cross-section ought to satisfy conditions

(1a) $$\begin{array}{cc} \int_{A_{w}}\;E_{w}\varepsilon \;dA+\int_{A_{r}}\;E_{r}\varepsilon \;dA & =N(=0), \end{array}$$

(1b) $$\begin{array}{cc} \int_{A_{w}}\;E_{w}\varepsilon z\;dA+\int_{A_{r}}\;E_{r}\varepsilon z\;dA & =M(=EI\kappa ), \end{array}$$

where *N*, *M*, and κ are the normal force, bending moment, and curvature in the cross-section, respectively; $A_{w}$ and $A_{r}$ refer to cross-sectional domains of wood and reinforcement, respectively; $E_{w}$ and $E_{r}$ are the moduli of elasticity of wood and reinforcement, respectively; ε denotes the axial strain; and *z* is the vertical coordinate of points of the domain in a suitable coordinate system. Strains ε are to be expressed parametrically in terms of the curvature and the position of the neutral axis in order to solve the equation system for an effective modulus of elasticity $E_{r}$ of the reinforcement, which is equivalent to the bending stiffness (EI) obtained from the measurements.

The analytically predicted behaviour may differ from the observations, which is readily reflected in possible discrepancies between calculated and nominal moduli of the CFRP. Differences may be attributable to the effectiveness of the preparation of in situ reinforcement with accidental damages or imperfections introduced during the procedure or inaccurate assessment of timber material properties.

Several authors reported a low or moderate increase in stiffness resulting from FRP reinforcement. In a number of cases, the measured and predicted values significantly differ, as shown in a review article [26]. Therefore, we propose to improve the modelling by considering different moduli for tension ($E_{t}$) and compression ($E_{c}$) in timber. (Note that different moduli were considered previously, e.g., by [35,36], for finite element simulations for knotted beams, as well as for the analysis of load-bearing capacities by [37].)

By tension and compression zones distinguished in terms of material properties, the resulting inhomogeneous cross-section should satisfy conditions

(2a) $$\begin{array}{cc} \int_{A_{t}}\;E_{t}\varepsilon \;dA+\int_{A_{c}}\;E_{c}\varepsilon \;dA & =N(=0), \end{array}$$

(2b) $$\begin{array}{cc} \int_{A_{t}}\;E_{t}\varepsilon z\;dA+\int_{A_{c}}\;E_{c}\varepsilon z\;dA & =M(=EI\kappa ), \end{array}$$

where, further to the previously introduced notation, subscripts *t* and *c* refer to tension and compression in timber, respectively, and *E* is the modulus of elasticity of an equivalent homogeneous cross-section. Tension and compression domains and strains are to be expressed parametrically with the position of the neutral axis. The solution of the equation system yields the formula

(3) $$E\;\left(\mathrm{MOE}\right)=4E_{t}E_{c}{\left(E_{t}^{1/2}+E_{c}^{1/2}\right)}^{-2},$$

which relates the tension and compression moduli via MOE.

Stress resultants in the cross-section are to be modified to account for the different moduli such that in Equations (1a) and (1b), the wood domain is divided into tension and compression zones:

(4a) $$\begin{array}{cc} \int_{A_{t}}\;E_{t}\varepsilon \;dA+\int_{A_{c}}\;E_{c}\varepsilon \;dA+\int_{A_{r}}\;E_{r}\varepsilon \;dA & =N(=0), \end{array}$$

(4b) $$\begin{array}{cc} \int_{A_{t}}\;E_{t}\varepsilon z\;dA+\int_{A_{c}}\;E_{c}\varepsilon z\;dA+\int_{A_{r}}\;E_{r}\varepsilon z\;dA & =M(=EI\kappa ). \end{array}$$

Assuming the modulus of the reinforcement to be nominal, the solution of the equation system (Equations (3), (4a), and (4b)) yields the wood moduli, which can constitute a more adequate representation of the composite system than the assumption of the homogeneous wood section. The obtained compression moduli can be compared with values obtained from experiments.

**Figure 3:**
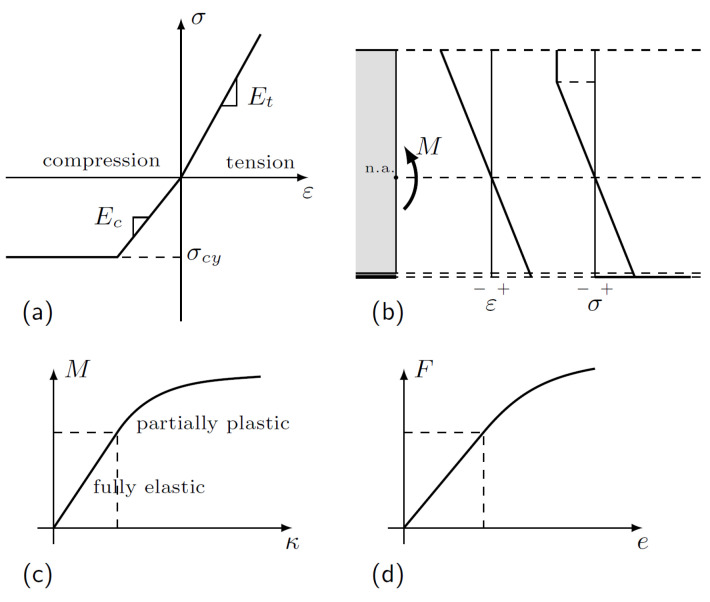
Constitutive modelling of timber cross-section. (**a**) Wood stress–strain relationship: elastic in tension, elastic–plastic in compression. (**b**) Strain and stress fields in the timber–CFRP composite cross-section. (**c**) Curvature–moment relationship based on the applied constitutive model and cross-section. (**d**) Simulated load–deflection curve based on the beam configuration and constitutive model.

Mechanical behaviour beyond the linear range is dependent on the non-linearity of timber in compression. Considering the commonly used constitutive model consisting of linear elasticity and perfect plasticity (Figure 3a), the stress–strain field in a cross-section can be reconstructed in terms of the curvature κ of the axis line of the beam (Figure 3b). The stress resultant yields the bending moment, establishing a bending moment–curvature relationship (Figure 3c), which then enables the simulation of the load–deflection diagram (Figure 3d) based on the applied beam configuration and model.

### 3.3. Finite Element Analysis

**Figure 4:**
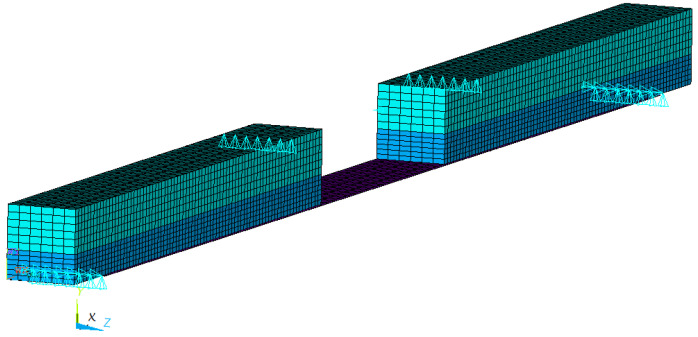
FEM model of a beam reinforced with CFRP (a section is removed for illustration purposes).

Finite element simulations were carried out to verify the analytical derivations based on the one-dimensional beam model. The three-dimensional FE models for both test groups (0 and F) were created in the general purpose software ANSYS (Canonsburg, PA, USA). The finite element mesh of a reinforced beam is shown in Figure 4. Supports and loadings are defined at appropriate nodes indicated by turquoise triangles. Particularly, to create symmetric simple supports, nodes along the support lines are constrained vertically and along one of them also transversely. Longitudinal support is provided by constraining a node centred on the top face in the longitudinal direction.

In structural modelling, wood is considered a linearly elastic orthotropic material at low-stress levels. While it is typically assumed that elasticity is preserved until brittle failure in tension, some kind of non-linear behaviour is adopted beyond elastic limit stress or yield stress in compression. This can be perfect plasticity, multilinearity, higher-order stress–strain relationships, etc.

In this study, elastic behaviour is characterised by the general form of Hooke’s law with nine orthotropic constants in the material stiffness matrix. Moduli parallel to grain were taken from the computation of this work, while the rest of the constants were adopted from literature [4,5,38,39].

Non-linearity was effectuated by the generalised anisotropic Hill potential model incorporated in ANSYS, which allows the definition of distinct yield strengths in tension and compression and different behaviour in the principal material directions. An anisotropic work-hardening rule and an associated flow rule are included in the model. The material behaviour is characterised by the stress–strain curves in the three orthogonal and shear directions. For each direction, a bilinear response is considered. Plasticity is represented by zero tangent modulus at the compression yield stress and the absence of plasticity in tension by sufficiently large values for yield stress. The elastic moduli of the material are the same for compression and tension. In order to implement different values, the timber domain was divided into a tension part and a compression part such that the appropriate modulus is defined in each.

The composite reinforcement is a mixture of epoxy matrix and fibres forming a transversely isotropic material. Epoxy is a linearly elastic isotropic material with modulus $E_{m}=4.5$ GPa and Poisson’s ratio ν=0.3. The modulus of elasticity for the fibres is provided by the manufacturer. Assuming that the fabric has a maximum possible compactness at its nominal thickness (volume ratio f≈0.9), the longitudinal ($E_{1}$) and transverse ($E_{2}$) moduli of the composite are obtained by the rule of the mixture and the inverse rule of mixture, respectively, as

(5) $$E_{1}=E_{f}f+E_{m}\left(1-f\right),\;E_{2}={\left(\frac{f}{E_{f}}+\frac{1-f}{E_{m}}\right)}^{-1}$$

where subscripts *f* and *m* refer to fibre and matrix, respectively. The shear modulus $G_{12}$ and the Poisson’s ratio $\nu_{12}$ are approximated with the rules as

(6) $$G_{12}={\left(\frac{f}{G_{f}}+\frac{1-f}{G_{m}}\right)}^{-1}$$

and

(7) $$\nu_{12}=\nu_{f}f+\nu_{m}\left(1-f\right).$$

Poisson’s ratio $\nu_{21}=E_{2}/E_{1}\cdot \nu_{12}$ follows from the symmetry of the material matrices.

Three-dimensional solid elements (SOLID45) were employed to represent the wood material. The eight-node element has three translational degrees of freedom per node and is compatible with the defined Hill anisotropic model. A fine mesh was achieved by dividing the beam into 200 elements longitudinally (*x*-direction), and 12 and 6 elements in the other two directions (*y* and *z*, respectively). By constraining the displacements of respective nodes, conditions of the test arrangement were realised (simple supports at the bottom face and two lines of loads at the top).

Three-dimensional Layered Structural Solid Elements (SOLID185) were used to represent the unidirectional CFRP composites and epoxy. This element can be used to create multilayered thick shells or solids, and the anisotropic material characteristics are included in the element input data. The element needs to be associated with predefined shell sections for both the FRP and the epoxy with their specifications (thickness, material properties, and orientation). The CFRP is relatively thin; so, the mesh had to be generated carefully to comply with rules on aspect ratios and to avoid improperly shaped elements for adequate accuracy. The longitudinal lines within the volume representing the CFRP were divided into certain sub-lines. The vertical ones along the CFRP thickness were divided into two sub-lines and the mesh was later defined accordingly.

No constraint equations were defined to link the wood and CFRP components since both solid elements had the same degrees of freedom. Interface components and slide behaviour were not considered as an adequate bond between the wood and the CFRP was assumed.

## 4. Results

### 4.1. Stiffness

**Figure 5:**
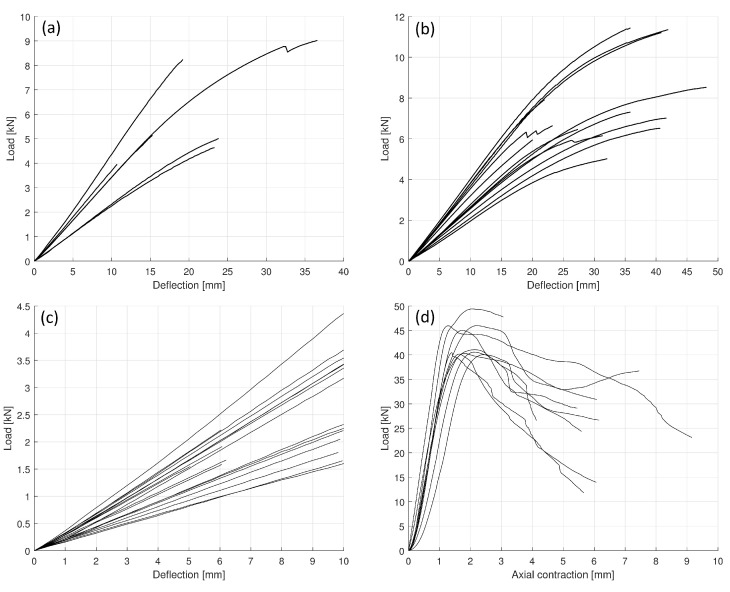
Load–displacement curves from all tests. (**a**) Load–deflection curves of non-reinforced specimens (group 0) from bending tests until failure. (**b**) Load–deflection curves of beams reinforced with carbon-fibre fabrics (group F) from bending tests until failure. (**c**) Load–deflection curves for all specimens without reinforcement from bending tests in the elastic range. (**d**) Load–displacement curves from axial compression tests.

Load–displacement curves of all tests are displayed in Figure 5. Load–deflection curves of specimens tested for bending until failure are shown in Figure 5a,b for non-reinforced and reinforced beams, respectively. Curves from elastic loading of group F prior to reinforcement together with the linear sections of curves for group 0 are shown in Figure 5c. Load–displacement curves from axial compression tests are plotted in Figure 5d.

Specimen data of all test groups are summarised in Table 2. Linearly elastic behaviour of pure timber (without reinforcement) is characterised by the slopes (SN) obtained from linear curve fitting to load–deflection curves. In the case of group 0, the linear section of the complete curves was analysed as input, while in the case of group F, curves from elastic loadings prior to reinforcement were used. Based on the geometric properties, the modulus of elasticity of wood ($E_{w}$) was computed by the classical Euler beam model. Mean values and standard deviations were computed (see Table 3) with two-sample *t*-tests showing that the samples can be regarded as coming from the same distribution at significance level p=0.05. However, the data show considerable scattering, highlighting the importance of obtaining specimen-specific data for the subsequent analysis. (Note that the variation of the modulus has previously been assumed to have a connection with the density of the wood, see e.g., [34], particularly as higher density implies higher modulus, just as it is apparent in Table 2).

Specimens in group F were tested until failure after the reinforcement was applied, and the linear section of the load–deflection curves was used as input for curve fitting. The obtained slopes (SR) are shown in Table 2 together with the increment with respect to the non-reinforced values in percentages (Δ).

**Figure 6:**
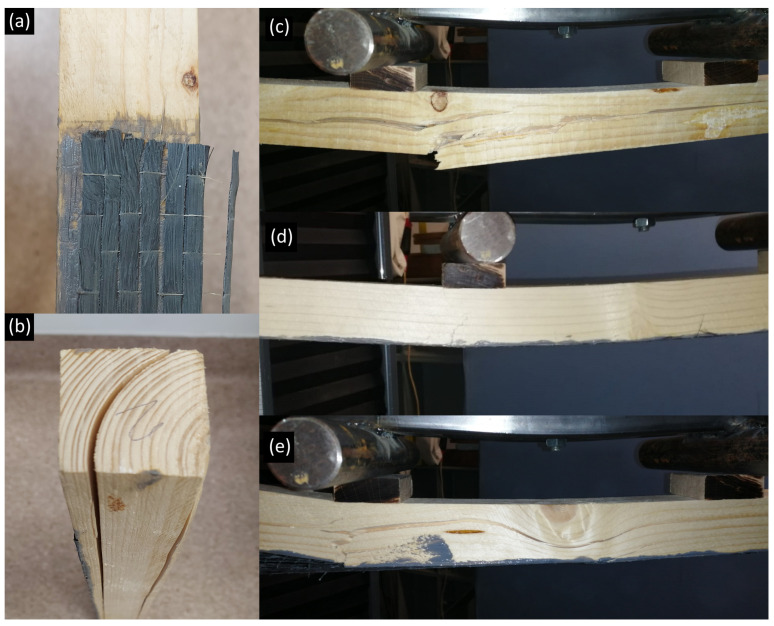
Failure. (**a**) Defected specimens with reinforcement slipping. (**b**) Defected specimens with cylindrical crack. (**c**) Non-reinforced specimen, tension failure. (**d**) Reinforced specimen, formation of a crack in the tension zone. (**e**) Reinforced specimen, fibre deviation around a knot and crack in the tension zone.

In group F (fabric reinforcement), it was found that three specimens produced exceptionally low increases (only up to 7%), which was contradictory to prior expectations. Inspection revealed that one of these specimens suffered a failure in the bond between the timber and fabric, as apparent from the slip of the reinforcement at one end of the beam both in the longitudinal and transverse directions leading to insufficient exertion of the capacities of the reinforcement, see Figure 6a. In the case of another specimen, a long cylindrical crack surface developed along a growth ring stretching along half the span of the beam, see Figure 6b, which obviously weakened the cross-section. The crack was unfortunately invisible during the test as the load pressed the detached surfaces together. The last of the three specimens suffered a splintering failure, which disabled the identification of any possible visible cause of the problem.

The rest of the specimens did not exhibit any unexpected behaviour producing stiffness increments in the range of approx. 13% to 30%. The bending stiffness EI of the reinforced beams calculated from the slopes and the modulus of the wood material obtained from the elastic testing were used in Equations (1a) and (1b) to calculate the hypothetical modulus of the reinforcement ($E_{r}^{*}$). In the case of a perfect theoretical scenario, the calculated values would coincide with the given nominal moduli (230 GPa). The results are grouped into three categories. The three faulty specimens unsurprisingly produced nonsensical results (very low or even negative values); some specimens were very close to theoretical expectations (approx. 200 GPa to 230 GPa), and the rest fell short of the expectation to some degree (approx. 150 GPa to 200 GPa). Since these specimens showed no sign of any defect in the wood material or the reinforcement and effort had been made to carefully prepare the in situ reinforcement, the deficiency prompted enhancement of the analytical models.

### 4.2. Tension and Compression Moduli

Assuming nominal reinforcement behaviour and different wood moduli in the tension and compression, the solution of the equation system (Equations (3), (4a), and (4b)) for the wood moduli ($E_{c}$ and $E_{t}$) are shown in Table 2. The defected specimens (specimens F-3, F-4, and F-5) were excluded from the analysis. In the case of specimen F-1, there is a significant difference between the tension and compression, which coincides with the occurrence of a low increase in stiffness (the lowest of all non-defected specimens). For the rest of the specimens, the difference between the moduli is relatively small, indicating that the consideration of different behaviour in compression and tension with a reasonable tolerance leads to good compliance with the expected nominal properties of the reinforcement.

The calculated compression moduli are compared with the results of compression tests presented in Figure 5d. The mean values and standard deviations are shown in Table 4. A two-sample *t*-test showed that the samples possess equal means at significance level p=0.05.

### 4.3. Load-Bearing Capacity and Failure

Load–deflection curves shown in Figure 5 are obtained from displacement-controlled measurements. The load-bearing capacity and the ultimate deflection were determined at the instance when the maximum load was attained, disregarding the rest of the measurement. (In the case of a few specimens, minor drops were observed in the curves, which were considered only as local maxima if an increase in load-bearing could be reached at higher deflections).

Contrary to the analysis of stiffness, load-bearing capacity and ultimate deflection cannot be determined for both non-reinforced and reinforced configurations of a beam (because of its obvious destructive nature); instead, two different samples (0 and F) are compared statistically. Mean values and standard deviations in both groups and increase by reinforcement with respect to the non-reinforced group are given in Table 5. Despite the amount of reinforcement being small (0.268–0.307% in volume fraction depending on the exact dimension of the beams), a significant increase in load-bearing capacity (27%) and ultimate deflection (53%) was achieved. Attention was paid to preparing the reinforcement in situ as accurately as possible (with one failed specimen, as mentioned before), proving that the preparation technique is important and, thus, the capabilities of the reinforcing fibres can be exploited.

All specimens in the non-reinforced group failed in tension with the splitting of fibres parallel to the grain. The tension zone was free from knots to avoid the loss of capacity they may potentially cause. (Note that if the beams contained knots, they were oriented to be located in the compression zone.) The failure originated in weak points in the tension zone, and the fracture of the fibres is usually followed by horizontal cracks propagating towards the ends parallel to the grain. Most specimens exhibited insignificant ductility, suggesting that the tensile failure took place before the plastic capacity of wood in compression could be utilised. In the case of one specimen, the ductility was not negligible; this specimen had the highest load-bearing capacity and potentially the highest tensile strength, allowing for some plasticity in the compression zone to develop.

With a few exceptions, the reinforced specimens had significantly larger ultimate displacement and ductility than the reference group. This supports the purpose of reinforcement to attract tensile stresses in order to facilitate the exploitation of both the tension and compression capacities of timber. Most of the specimens underwent moderate deformation in compression before sudden tension failure. One specimen behaved differently as it contained a knot. Even though it was located in the compression zone of the timber, the disturbed fibre paradigm with intense fibre curvature reached into the tension zone. It caused stress concentration that initiated the failure in the tension zone, though the effect of its contribution cannot be assessed. A few failure cases are illustrated in Figure 6c–e.

In an experimental study involving similar reinforcement of the same wood species [12], it was also found that the predominant failure mode was timber rupture in tension. Other modes occurred only occasionally; for example, shearing near supports if the shear strength of the specimen was too low, compression failure parallel to grain, or the tensile failure of the reinforcement if it was not prepared at the required quality. In the current work, apart from the few extraordinary cases mentioned in Section 4.1, only tensile failures were observed.

Compliance, the energy absorption measure of the specimens, can also be computed to quantify the load–deflection behaviour. It is numerically evaluated as the area covered under the curve in the diagram. The increase in mean compliance in the reinforced group F compared with the non-reinforced reference group 0 was 133%, see Table 5.

### 4.4. Numerical Simulations

**Figure 7:**
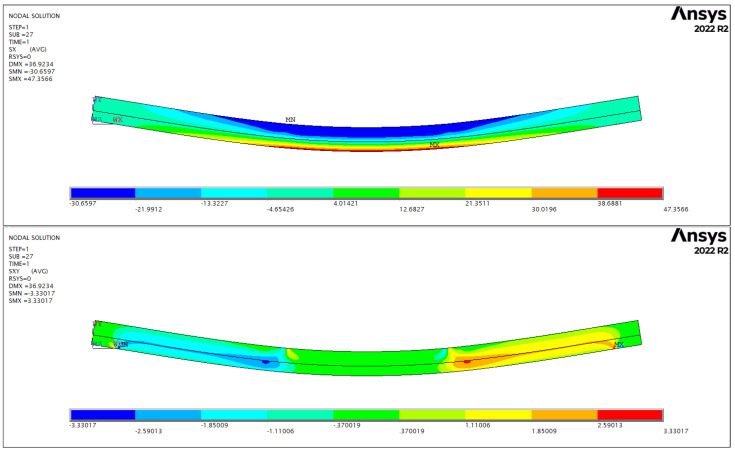
Longitudinal normal stress (**top**) and shear stress (**bottom**) distributions for specimen F-6.

Analysis of the entire load–deflection behaviour, including the non-linear part, was carried out using both the one-dimensional beam model and the three-dimensional finite element simulation. The former incorporates non-linearity with respect to material plasticity through the moment–curvature relationship, while the latter implements a complete non-linear analysis in all aspects (geometry, material). The longitudinal normal stresses are dominant in the orthotropic stress fields. Figure 7 illustrates the stress distribution in the case of one of the specimens. The stresses are the largest in the middle section of the beam ranging from extreme tension at the bottom to extreme compression at the top, in correspondence with the one-dimensional beam model. The most typical failure mode, as shown by the experiments, is timber rupture in tension. It is verified by the results of the simulations since stresses in transverse directions as well as in shearing are not significant in magnitude compared to their respective strength characteristic to spruce.

**Figure 8:**
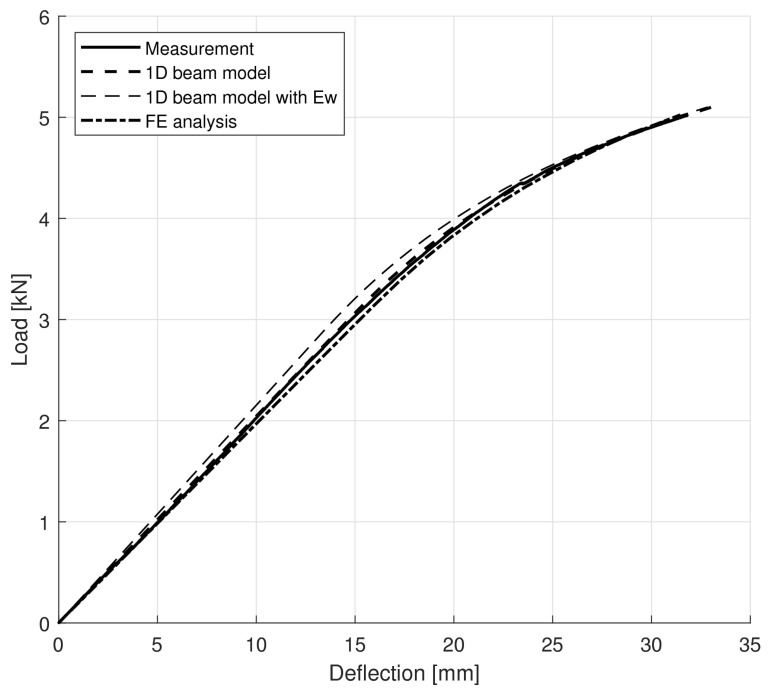
Load–deflection curves for specimen F-6. Heavy solid, dashed, and dash-dotted lines refer to measurements, 1D simulation results, and FE simulation results, respectively. For comparison, beam model calculations with a single wood modulus $E_{w}$ are shown by a thin dashed line.

For each specimen, moduli obtained for tension and compression were applied. The difference between the simulated and measured load–deflection curves can be quantified by the sum of least squares, and the plastic yield stress in the simulations was set to result in the minimum error, which could be easily achieved with the required precision after a few trials. One particular specimen (F-6) is shown for illustration in Figure 8.

Both simulated curves fit to the measurements very closely, with the result of the finite-element analysis being slightly softer as it is less rigid than the classical beam model. The mean difference in stiffness (slope of the linear part of the curves) between the finite element results and the measurements is 6.01%. The simulated curves indicate that with the chosen models, not only were the linear parts captured but the non-linearity could be properly modelled as well. The obtained compression yield stresses can also be compared with the results of the direct axial compression tests shown in Figure 5d. Considering plasticity at the maximum load level, the plastic yield stresses can be simply obtained. Table 6 gives brief statistics on the yield stresses from measurements and simulations. Mean values in the one-dimensional and finite element computations are close to the test result with errors of 6.84% and 1.55%, respectively.

For comparison, beam model calculations with a single wood modulus $E_{w}$ are also shown in the diagram by a thin dashed line. It is apparent that negligence of the distinction between tension and compression overestimates the stiffness of the beam.

It is clear that the mean values are in good correspondence, which indicates that the inversely calculated values are adequately verified by measurements.

The match between the simulated and the measured load–deflection curves can also be given by other quantities. The relative difference between the ultimate loads at the ultimate displacement had a mean value of only 0.14%. Compliance, the energy absorption capacity of the specimen, is another characteristic of the curves, numerically evaluated as the area covered under the curve. The mean of the relative differences is found to be 0.61%.

Note that even though the measured and simulated load–deflection curves are in good correspondence, the differences may further be reduced by applying more sophisticated non-linear models (e.g., tri-linear stress–strain diagrams suggested by Hill, etc.), though it requires more material parameters to achieve.

## 5. Conclusions

Over the last few decades, several studies reported on the mechanical enhancement of timber by applying fibre-reinforced polymers. The increase in stiffness observed in experiments covered a wide range with a minimum near zero and often clearly contradicted expectations by analytical models. This work presented an experimental, analytical, and numerical study on timber beams reinforced with CFRP to provide better modelling for the explanation of the discrepancies. The experiments featured measurements of stiffness of beams under bending, with and without CFRP reinforcement of each producing specimen-specific data, which enabled the direct analysis of stiffness increase on an individual basis. The role of fibre-reinforced fabric in the analysis is significant because the composite structure enables the distinction between tension and compression moduli as opposed to a pure timber structure in which the moduli cannot be uniquely determined. The main findings of the work are summarised as follows:

- The adequate in situ reinforcement of non-defected timber beams produced an increase in stiffness in the range 13% to 30%, even with a small amount (0.268% to 0.307% volume fraction) of fibre (disregarding the failed specimens). Generally, the beams exhibited lower stiffness than expected by analytical or numerical modelling, and the differences could not be attributed to faulty or imperfect reinforcement preparation.
- Computations assuming different tension and compression moduli for timber showed in most cases that measured stiffness increase could be achieved using the nominal factory value for reinforcement modulus. Both the beam model and the finite element simulations using three-dimensional models showed good correspondence with the measured data with appropriately set compression yield stresses, indicating that the applied models are adequate for the description of the non-linear behaviour as well. The finite element simulations of the load–deflection curves resulted in an average error of 0.14% in ultimate force and 0.61% in compliance. The calculated compression moduli are verified by direct compression test results (mean values of 8.682 GPa and 7.016 GPa, respectively, with *t*-test showing statistical match at p=0.05). The yield stresses are also validated by the results of the axial compression tests (error of 6.84% for the beam model and 1.55% for the finite element model with respect to compression tests).
- Statistical comparison of stiffness values in the reinforced group with the non-reinforced reference group showed that they come from the same sample. The average increases of 27% in load-bearing capacity, 53% in ultimate displacement, and 133% in compliance were achieved. These indicate that both tension and compression (with plasticity) capacities of wood could be effectively exploited and that the reinforcing technique performed adequately (with the exception of one reported case).
- The results lead to the conclusion that the differences observed between experiments and analytical considerations regarding the bending stiffness of reinforced timber beams are likely to be attributed to the use of a single modulus in timber for tension and compression, and the distinction enables adequate modelling complying with experiments.

Differences between simulated and experimentally obtained stiffness increase are in most cases in favour of the calculations, which would therefore overestimate the stiffness of the reinforced beams in a design process. This, in turn, would lead to non-compliance with the objectives on structural deflections at the expense of safety. Its effect is likely to rise if larger amounts of reinforcement (in volume ratio) are applied. Inclusion of this distinction into modelling could more efficiently describe the mechanical behaviour of timber with or without reinforcement, and enable designers to apply the right amount of reinforcement for their task.

The research can be continued with experimental investigation of beams with different dimensions and amount of reinforcement; however, attention needs to be paid to eliminate all factors that could invalidate calculations, e.g., imperfections of in situ reinforcement, insufficient bond contact if factory-product lamellae are used, defects of timber, etc.

## Figures and Tables

**Table 1:** Specifications of test groups for bending.

Group	Reinforcement	Amount	Number	Test Type
0	none	-	6	total
F	fabrics	full span	13	elastic + total

**Table 2:** Summary of stiffness and moduli of bending test specimens in groups of non-reinforced beams (0) and beams reinforced with CFRP fabric (F). Slopes of the load–deflections curves are obtained by linear regression in the linear elastic range for all groups in the non-reinforced stage (SN). Slopes for the reinforced group after reinforcement are given by SR with increment Δ. Calculated moduli of elasticity for the wood are denoted by $E_{w}$, and the hypothetical moduli of the reinforcement (assuming the calculated $E_{w}$) by $E_{r}^{*}$. Cross-sectional height (*h*) and width (*b*) as well as wood density (ρ) are also shown. Empty cells indicate data not applicable. Dashes (–) are displayed where results are not computed due to measurement faults.

Sp.	SN ($\frac{\mathrm{kN}}{\mathrm{mm}}$)	SR ($\frac{\mathrm{kN}}{\mathrm{mm}}$)	Δ (%)	$E_{w}$ (GPa)	$E_{r}^{*}$ (GPa)	$E_{c}$ (GPa)	$E_{t}$ (GPa)	*h* (mm)	*b* (mm)	ρ ($\frac{g}{{\mathrm{cm}}^{3}}$)
0-1	0.2240			6.042				47.0	47.0	0.3761
0-2	0.4285			12.972				45.4	46.2	0.5252
0-3	0.2278			6.052				46.5	48.4	0.3725
0-4	0.3594			9.743				46.9	47.4	0.4669
0-5	0.3347			8.661				47.9	45.8	0.4046
0-6	0.2262			7.450				46.0	42.8	0.5446
F-1	0.2635	0.2977	12.95	7.063	81.388	4.286	13.756	47.2	47.9	0.3552
F-2	0.3114	0.3667	17.75	8.063	149.175	6.246	10.802	48.2	46.4	0.3617
F-3	0.2700	0.2729	1.06	7.174	−18.099	–	–	47.7	46.8	0.3457
F-4	0.3730	0.3992	7.05	10.825	59.884	–	–	46.3	46.9	0.5275
F-5	0.3195	0.3323	3.99	8.974	14.098	–	–	47.0	46.3	0.4418
F-6	0.1592	0.2015	26.63	5.734	148.412	4.718	7.117	44.0	44.0	0.5262
F-7	0.1832	0.2382	29.98	6.314	198.263	6.328	6.295	45.0	43.0	0.5180
F-8	0.3595	0.4109	14.29	13.271	177.016	11.421	15.607	43.0	46.0	0.6020
F-9	0.1658	0.2162	30.38	8.136	232.285	9.387	7.115	40.0	43.0	0.5190
F-10	0.2080	0.2664	28.09	8.214	231.744	9.496	7.174	43.0	43.0	0.5237
F-11	0.3257	0.3796	16.52	13.192	202.734	14.160	12.313	42.0	45.0	0.5986
F-12	0.3381	0.3925	16.07	12.761	196.224	13.176	12.358	43.0	45.0	0.6443
F-13	0.2223	0.2732	22.91	8.007	180.839	7.601	8.446	44.0	44.0	0.5256

**Table 3:** Summary of modulus of elasticity of wood in both groups.

MOE ($E_{w}$) (GPa)	Mean	Standard Deviation
0	8.487	2.634
F	9.143	2.701

**Table 4:** Statistical overview of wood compression moduli from calculations and measurements.

Compression Moduli (GPa)	Mean	Standard Deviation
Calculations (Equations (3), (4a), and (4b))	8.682	3.441
Compression tests	7.016	1.032

**Table 5:** Mean values and standard deviations of the maximum loads, deflections, and compliance in all groups, and increase with respect to non-reinforced group.

Group	Max Load (kN)	Increase	Max Def. (mm)	Increase	Compliance (Nm)	Increase
	Mean (st. dev.)	(%)	Mean (st. dev.)	(%)	Mean (st. dev.)	(%)
0	6.002 (2.095)	-	21.480 (8.912)	-	77.247 (63.899)	-
F	7.623 (2.163)	+27.01	32.877 (9.799)	+53.06	180.443 (82.026)	+133.59

**Table 6:** Average values of the compression yield stress (MPa), and standard deviations.

Compression Yield Stress	Mean	Standard Deviation
1D analysis	43.20	15.20
FE analysis	45.65	17.11
Compression tests	46.37	3.56

## Data Availability

Not applicable.
